# Effluent from ischemic preconditioned hearts confers cardioprotection independent of the number of preconditioning cycles

**DOI:** 10.1371/journal.pone.0243220

**Published:** 2020-12-03

**Authors:** Katharina Feige, Annika Raupach, Carolin Torregroza, Jan Muehlenbernd, Martin Stroethoff, Sebastian Bunte, Markus W. Hollmann, Ragnar Huhn

**Affiliations:** 1 Department of Anesthesiology, University Hospital Duesseldorf, Duesseldorf, Germany; 2 Department of Internal Medicine, Elbe Clinics Stade-Buxtehude, Stade, Germany; 3 Department of Anesthesiology, Amsterdam University Medical Center (AUMC), Amsterdam, The Netherlands; Virginia Commonwealth University, UNITED STATES

## Abstract

Coronary effluent collected from ischemic preconditioning (IPC) treated hearts induces myocardial protection in non-ischemic-preconditioned hearts. So far, little is known about the number of IPC cycles required for the release of cardioprotective factors into the coronary effluent to successfully induce cardioprotection. This study investigated the cardioprotective potency of effluent obtained after various IPC cycles in the rat heart. Experiments were performed on isolated hearts of male Wistar rats, mounted onto a Langendorff system and perfused with Krebs-Henseleit buffer. In a first part, effluent was taken before (Con) and after each IPC cycle (Eff 1, Eff 2, Eff 3). IPC was induced by 3 cycles of 5 min of global myocardial ischemia followed by 5 minutes of reperfusion. In a second part, hearts of male Wistar rats were randomized to four groups (each group n = 4–5) and underwent 33 min of global ischemia followed by 60 min of reperfusion. The previously obtained coronary effluent was administered for 10 minutes before ischemia as a preconditioning stimulus. Infarct size was determined at the end of reperfusion by triphenyltetrazoliumchloride (TTC) staining. Infarct size with control effluent was 54±12%. Effluent obtained after IPC confers a strong infarct size reduction independent of the number of IPC cycles (Eff 1: 27±5%; Eff 2: 35±7%; Eff 3: 35±8%, each P<0.05 vs. Con). Effluent extracted after one cycle IPC is comparably protective as after two or three cycles IPC.

## Introduction

Ischemic preconditioning (IPC) still remains the strongest cardioprotective stimulus inducing significant infarct size reduction [1]. The protective effect of IPC—regularly practiced with three cycles of five minutes of myocardial ischemia and reperfusion—is not limited to the heart but also feasible at other organs [2]; a phenomenon called remote ischemic preconditioning (RIPC) [3]. This demonstrates that a release of humoral factors induced by an ischemic stimulus, transferred via the blood to distant organs is able to confer protective properties. Profound research has revealed that these humoral factors—in form of effluent or plasma—can be transferred from a donor heart after preconditioning to a naive heart, as an inter-heart transfer [4, 5]. This transfer of humoral factors was shown between different animal species but also in a translational, clinical approach; plasma containing protective humoral factors from volunteers conferred cardioprotection in the isolated rat heart [6]. Serejo et al. demonstrated that pooled effluent collected in the reperfusion phases during IPC consisting of three cycles of five minutes of myocardial ischemia and reperfusion in the isolated rat heart reduced infarct size when transferred to naive hearts [7]. As yet, nothing is known about the release of humoral factors to the effluent of IPC treated hearts and the cardioprotective potency after every single IPC cycle. Furthermore, the interrelation between the cardioprotective properties in terms of infarct size reduction, release of humoral factors and the amount of the required IPC stimuli remains to be determined. The present study aims to investigate whether the number of IPC cycles influences the release of humoral cardioprotective factors into the coronary effluent. We hypothesize that the infarct size reducing effect of the coronary effluent depends on the number of conducted IPC stimuli.

## Materials and methods

The present study was conducted in accordance with the Guide for the Care and Use of Laboratory Animals published by the National Institutes of Health (Publication number 85–23, revised 1996) and was performed after obtaining approval from the Animal Ethics Committee of the University of Duesseldorf, Germany. The animals were obtained from the breeding facility at the Central Animal Research Facility of the Heinrich-Heine-University Duesseldorf. Experiments were conducted and results were reported in accordance with the ARRIVE guidelines.

### Surgical preparation

Surgical preparation was performed as described previously [8]. In brief, male Wistar rats were anesthetized by intraperitoneal injection of pentobarbital (80 mg/kg body weight). Hereafter, animals were thoracotomized for the removal of the hearts. The hearts were mounted on a Langendorff system and perfused with Krebs-Henseleit solution, enriched with 95% O_2_ and 5% CO_2_. The solution contains (in mM): 118 NaCl, 4.7 KCl, 1.2 MgSO_4_, 1.17 KH_2_PO_4_, 24.9 NaHCO_3_, 2.52 CaCl_2_, 0.5 EDTA, 11 glucose and 1 lactate at 37°C. For the duration of the experiments a constant pressure (80 mmHg) and temperature (37°C) was maintained. We inserted a fluid filled balloon into the left ventricle and kept an end-diastolic pressure of 2–8 mmHg. The hearts underwent an equilibration period of 20 minutes. We measured heart rate, left ventricular end-systolic pressure (LVESP), coronary flow and left ventricular end-diastolic pressure (LVEDP) continuously and digitized it at a sampling rate of 500 Hz by use of an analogue to digital converter system (PowerLab/8SP, ADInstrument Pty Ltd, Castle Hill, Australia). Left ventricular developed pressure (LVDP) was calculated as LVESP—LVEDP. Data were continuously recorded on a personal computer using Chart for Windows v5.0 (ADInstruments Pty Ltd, Castle Hill, Australia).

### Experimental protocol—Effluent sampling

**Fig 1:**
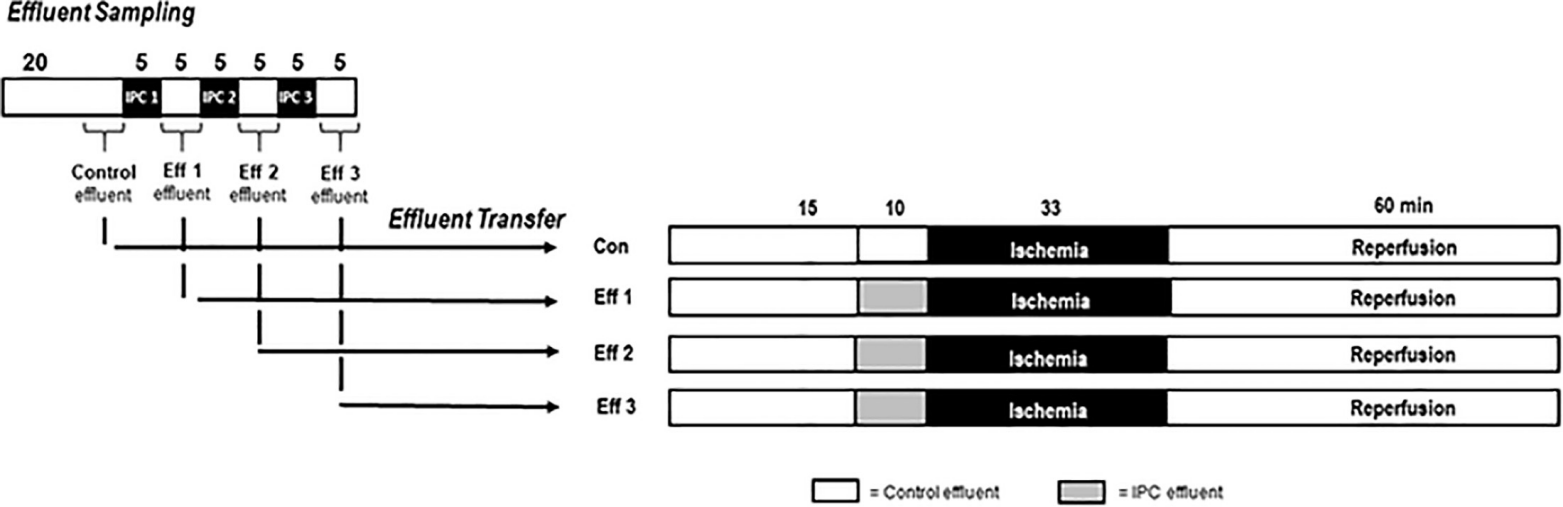
Effluent sampling and transfer protocol. Part 1: Effluent sampling. Coronary effluent was collected before (Con effluent) and during reperfusion after each 5-minute IPC cycle (Eff 1, Eff 2 and Eff 3). Part 2: Effluent transfer. Con = Control; Eff 1 = Effluent after one cycle of IPC; Eff 2 = Effluent after two cycles of IPC; Eff 3 = Effluent after three cycles of IPC; IPC = Ischemic preconditioning.

In a first part, coronary effluent was obtained at different time points as shown in Fig 1, part 1: effluent sampling (n = 5, at each time point). All hearts underwent 20 minutes of adaption period. For control group (Con), effluent was collected for 5 minutes after 15 minutes of adaption period, no IPC maneuver was performed. Effluent 1 (Eff 1) was collected for 5 minutes during the reperfusion period after the first cycle of IPC (IPC 1) before the second IPC cycle was performed. Effluent 2 (Eff 2) was obtained respectively in the following 5 minutes of reperfusion after the second cycle of IPC (IPC 2) maneuver. Coronary effluent for group Eff 3 was collected after the third cycle of 5 minutes IPC (IPC 3). In summary, 4 samples of effluent were collected from each heart, without (Con) and with IPC and reperfusion (Eff 1–3). For each heart, effluent was collected over a total of 5 minutes.

### Experimental protocol—Effluent transfer

In a second part, naïve rat hearts were randomly assigned to four groups (n = 4–5 per group, Fig 1, part 2: effluent transfer) and underwent the following protocol:

**Control (Con)**. Hearts received control effluent (no IPC maneuver performed) for 10 minutes before ischemia (infusion rate 1% of coronary flow (CF)).
**Effluent group 1 (Eff 1)**. Hearts received effluent, previously obtained from hearts in part 1 after 1 cycle IPC, for 10 minutes before ischemia (infusion rate 1% of CF).
**Effluent group 2 (Eff 2)**. Hearts received effluent, previously obtained from hearts in part 1 after 2 cycles IPC, for 10 minutes before ischemia (infusion rate 1% of CF).
**Effluent group 3 (Eff 3)**. Hearts received effluent, previously obtained from hearts in part 1 after 3 cycles IPC, for 10 minutes before ischemia (infusion rate 1% of CF).

After 60 minutes of reperfusion, hearts were cut into transverse slices, starting from the cardiac apex to just before the cardiac valvular plane. The slices were stained with 0.75% triphenyltetrazoliumchloride (TTC) solution. The size of the infarcted area was determined by planimetry using SigmaScan Pro 5^®^ computer software (SPSS Science Software, Chicago, IL) by a blinded investigator.

### Animal data

In Table 1 body weight, wet weight as well as time and level of maximal ischemic contracture of the four groups are shown. In the respective groups Eff 2 and Eff 3, one heart per group was excluded from further analysis because it did not fulfill the qualitative characteristics (threshold values of heart rate and LVDP) for measurements with the Langendorff-system.

**Table 1:** Weights and ischemic contracture.

	body weight (g)	heart weight wet (g)	time of max. ischemic contracture (min)	level of max. ischemic contracture (mmHg)
Con	290 ± 7	1.18 ± 0.02	17 ± 1	55 ± 2
Eff 1	291 ± 40	1.27 ± 0.12	16 ± 3	56 ± 4
Eff 2	265 ± 13	1.16 ± 0.06	15 ± 1	60 ± 10
Eff 3	301 ± 10	1.19 ± 0.05	15 ± 2	72 ± 6

Data are mean±SD. Con = Control; Eff 1 = Effluent after one cycle of IPC; Eff 2 = Effluent after two cycles of IPC; Eff 3 = Effluent after three cycles of IPC.

### Statistical analysis

Sample size calculation using GraphPad StatMate^™^ (GraphPad Software, San Diego, CA, USA) revealed a group size of n = 5 for detecting a 25% mean difference and a standard deviation of 10% in infarct size (power 80%, α<0.05 (two-tailed)). Primary endpoint was the infarct size. Data are expressed as mean±SD. Infarct sizes were analyzed by one-way analysis of variance (ANOVA) followed by Dunnett’s post hoc test (GraphPad Software V7.01, San Diego, CA, USA). Comparisons of hemodynamics between groups or between different time points within a group were performed with a two-way ANOVA followed by Tukey’s post hoc test. Differences were considered statistically significant when P values were less than 0.05.

## Results

### Infarct size

**Fig 2:**
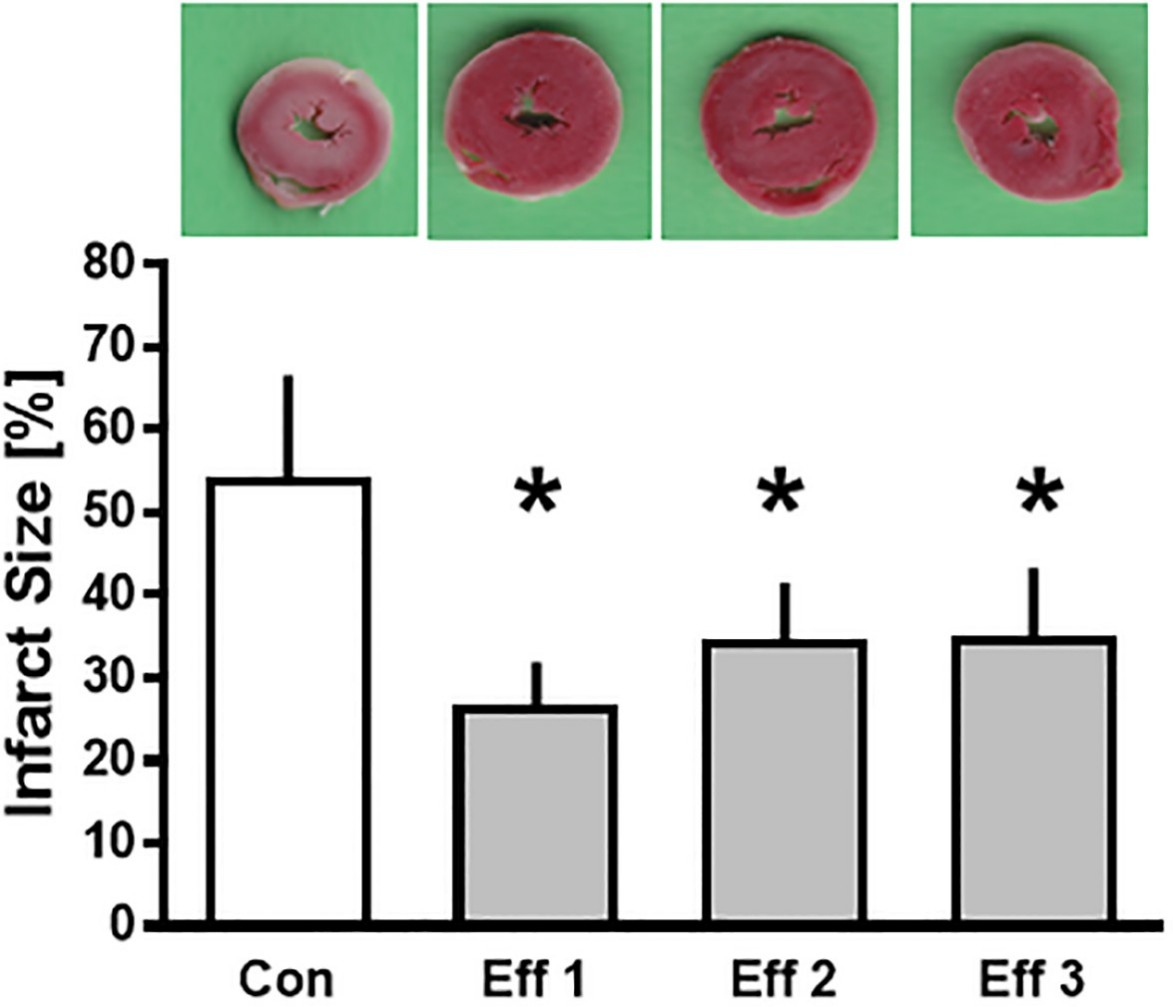
Infarct size measurement. Histogram shows the infarct size of controls (Con) and administration of effluent after IPC (Eff 1, Eff 2, Eff 3). Data are presented as mean±SD, *P<0.05 vs. Con. Representative TTC stained slices of the respective hearts.

Results from infarct size analysis are shown in Fig 2. In control hearts, infarct size was 54±12% (Fig 2). The administration of effluent significantly reduced infarct size independent of the number of IPC cycles compared to control (Eff 1: 27±5%; Eff 2: 35±7%; Eff 3: 35±8%, each P<0.05 vs. Con).

### Hemodynamic variables

Hemodynamic variables are summarized in Table 2. There were no differences in heart rate, LVDP and coronary flow between the groups. After ischemia and during reperfusion LVDP and coronary flow were statistically different from baseline in all groups.

**Table 2:** Hemodynamic variables.

	Baseline	PC	Reperfusion	
			30	60
*Heart rate (bpm)*				
Con	307 ± 29	288 ± 43	296 ± 73	254 ± 41
Eff 1	333 ± 70	330 ± 53	298 ± 48	236 ± 73
Eff 2	290 ± 46	305 ± 36	251 ± 44	229 ± 91
Eff 3	295 ± 24	278 ± 44	282 ± 36	272 ± 13
*LVDP (mmHg)*				
Con	121 ± 20	120 ± 20	19 ± 6*	27 ± 3*
Eff 1	123 ± 34	123 ± 22	37 ± 40*	39 ± 26*
Eff 2	139 ± 15	136 ± 13	39 ± 26*	22 ± 12*
Eff 3	155 ± 53	154 ± 27	14 ± 5*	18 ± 4*
*Coronary flow (ml/min)*				
Con	12 ± 1	11 ± 3	7 ± 1*	6 ± 1*
Eff 1	15 ± 3	14 ± 3	8 ± 2*	7 ± 2*
Eff 2	11 ± 2	10 ± 2	8 ± 2*	7 ± 2*
Eff 3	15 ± 3	15 ± 3	7 ± 2*	6 ± 2*

Data are mean±SD. Con = Control; Eff 1 = effluent after one cycle of IPC; Eff 2 = Effluent after two cycles of IPC; Eff 3 = Effluent after three cycles of IPC.

*P<0.05 vs. Baseline.

## Discussion

The present study investigated the cardioprotective properties of IPC-conditioned coronary effluent collected after various cycles of IPC from isolated buffer-perfused rat hearts then transferred to naïve hearts as a preconditioning stimulus. Our results indicate that a significant infarct size reducing effect of coronary effluent collected from IPC treated hearts is independent of the performed number of IPC cycles. Transferred effluent, taken after one cycle of ischemia and reperfusion confers infarct size reduction to the same extent as effluent taken after two or three cycles of ischemia and reperfusion.

Previous studies have demonstrated cardioprotective effects by pooled reoxygenated coronary effluent, collected in the intermittent reperfusion phases of several IPC cycles, transferred onto naïve hearts [4, 7, 9]. Dickson et al. were the first to detect infarct size reduction by administration of pooled effluent [4]. The used IPC protocol consisted of three cycles of five minutes of ischemia followed by ten minutes of reperfusion. Other studies confirmed the cardioprotective effect of pooled effluent with even reduced reperfusion phases of only five minutes [7, 9]. The transfer of pooled coronary effluent taken during IPC not only confers cardioprotection as a preconditioning stimulus, but also induces protection when administered at the onset of reperfusion as postconditioning [10]. Breivik et al. were able to demonstrate a significantly reduced infarct size compared to control when administering pooled IPC-effluent for 10 min at immediate reperfusion. However, in all above-mentioned studies, the coronary effluent was collected during reperfusion phases of all three cycles as a pooled effluent of ischemia and reperfusion and administered as a pooled, mixed effluent to a naïve heart. Hence, to this point it is unclear at which point of IPC cycles cardioprotective factors are released into the coronary effluent and whether all three cycles are needed to deliver these respective factors to confer infarct size reduction when administered as a conditioning stimulus. The current study now reveals—for the first time—that the effluent taken separately from each reperfusion phase have comparable cardioprotective potencies. Yet, the *ideal* IPC protocol—proposing exact number and duration as well as intensity of the required IPC cycles conferring the strongest cardioprotective effect—has still not been established [11]. A commonly used IPC protocol in the rat heart consists of two to four cycles of ischemia lasting for two to five minutes each, interspersed with reperfusion periods for two to five minutes [12]. Interestingly, Przyklenk et al. showed that even shorter periods of sublethal ischemia have induced significant infarct size reduction, while prolonged ischemic episodes (>15 minutes) are not potent in inducing cardioprotective effects [2]. In contrast, a single only 2-minute coronary occlusion was not effective as a preconditioning stimulus in the rabbit heart, presuming a stimulus’ intensity below the threshold. Next to the number of performed ischemia cycles, also the duration of complete restoration of blood flow after ischemia is of great importance with regard to triggering a preconditioning response [13]. A previous study suggested that a minimum reperfusion period of up to 1 min is required to confer cardioprotection [14]. While 1 minute of reperfusion induced significant infarct size reduction, 30 s of reperfusion as part of the IPC protocol was not effective. Hence, duration of reperfusion periods seems relevant in conferring cardioprotection by IPC. Short periods after sublethal ischemia are needed to sufficiently reintroduce oxygen into the cardiomyocyte, which is necessary to induce production of reactive oxygen species in the cell which in turn activates IPC signaling cascades [15].

The search of the *ideal* IPC protocol needs to also take into account possible confounding factors in patients, for example age and comorbidities such as diabetes mellitus type 2 (DM2). Schulman et al. demonstrated that increasing age imminently effects the amount of needed IPC cycles to induce cardioprotection [16]. In middle aged, *in vivo* hearts, three cycles of ischemia and reperfusion were mandatory to reduce infarct size whereas in young hearts IPC with only one cycle of ischemia and reperfusion was comparably protective. In aged hearts the cardioprotective effect of IPC was completely abrogated [16]. Recently we showed that plasma transfer from young healthy volunteers after IPC of the forearm with a blood pressure cuff to an aged rat heart *in vitro* significantly reduced infarct size after IR injury. Vice versa, plasma taken from aged healthy volunteers did not contain humoral factors with cardioprotective properties [6]. These data demonstrate that the release and/or the potency of cardioprotective humoral factors is influenced by age, but it also shows that plasma containing potent protective factors is able to reduce infarct size even under aged conditions. To our knowledge, while aging was shown to impact IPC, an influence of aging regarding effluent transfer has not been assessed so far. With regard to comorbidities and co-medications, until now there is no study examining the influence of these parameters on the protection by effluent. Interestingly, diabetes seems to impact IPC by possibly increasing threshold for protection. Yellon et al. demonstrated that in healthy rat hearts one, two and three cycles of IPC induced infarct size reduction, while in DM2 rats three cycles of IPC were necessary to confer cardioprotection [17]. Similar, Wider et al. showed that remote ischemic preconditioning reduced infarct size in healthy Zucker lean but not in Zucker fatty—a model for DM2 –rats [18]. Hence, an influence of comorbidities on effluent transfer is conceivable and needs further research.

In our study, we specifically collected the coronary effluent from young healthy rats without co-morbidities or co-medications excluding any of the above-mentioned confounding factors. Our results might indicate that under healthy conditions the release of humoral factors is maximal after a single cycle of ischemia and reperfusion and that the effect cannot be increased by additional stimuli. Still, the question remains open whether under diseased conditions the amount and/or type of humoral factors released are different or possibly even absent. Underlying mechanisms and released factors of myocardial conditioning by effluent transfer have been investigated; however only for pooled effluent. Maciel et al. [9] and others [7] showed that the protective factors released to the pooled coronary effluent after IPC are hydrophobic peptides with a size between 3.5 and 12 kDa. To this point the exact protective factors in the effluent have not been identified. However, a large number of signaling pathways in IPC transmitted cardioprotection are recruited at the cardiomyocyte sarcolemma through the activation of cell surface receptors by their endogenous ligands. This leads to triggering of several protective signaling cascades, ultimately targeting the mitochondria., especially the mitochondrial permeability transition pore (MPTP) and mitochondrial potassium channel (mK^+^) as integral player of cardioprotection [19–21]. Serejo et al. [7] suggested triggering of cardioprotective signaling pathways by effluent transfer via activation of protein kinase C (PKC) and ATP-sensitive potassium (K_ATP_)-channels [9]. Alongside, Breivik et al. demonstrated that the cardioprotective effect of coronary effluent applied after ischemia, as a postconditioning stimulus, was completely blocked when the Phosphoinositide 3-kinase (PI3K)-inhibitor wortmannin or the protein kinase B (Akt)-inhibitor SH-6 were administered [10]. PI3K and Akt belong to the reperfusion injury salvage kinase (RISK) pathway and PKC and K_ATP_-channels have been proven to be crucially involved in mediating the cardioprotective effect of different preconditioning strategies, for instance IPC [22]. The results of the above-mentioned studies indicate that protective factors in effluent from IPC treated animals transferred to naïve hearts leads to activation of the same cardioprotective signaling pathways as those triggered by direct IPC.

Our data are purely descriptive and are limited to research of the underlying mechanism which was beyond the scope of our study. The aim of our study, as a first approach, was to elucidate a possible difference in the cardioprotective effect of coronary effluent extracted after various numbers of IPC cycles. In contrast to previous studies, we have specifically focused our point of interest on the infarct size reducing effect of the respective non-pooled effluent after each cycle of IPC. A next step for future studies, could be investigating whether the amount or type of factors released into the effluent differ regarding the number of applied IPC cycles. Furthermore, more underlying mechanisms of cardioprotection by effluent transfer need to be identified, especially in the context of confounding factors, such as age. Recently, we demonstrated that mitochondrial large-conductance calcium-sensitive potassium (mBK_Ca_) channels are crucially involved in protecting aged myocardium against consequences of ischemia-reperfusion injury. While for many pharmacological and ischemic conditioning strategies, cardioprotective properties are reduced or even lost in the aged heart, activation of mBK_Ca_ channels still initiates significant infarct size reduction independent of age [23]. Hence, with regard to the influence of age and diseased myocardium, investigating the role of mBK_Ca_ in myocardial conditioning with effluent after IPC are of further interest and planned for future studies.

## Conclusions

Our data supports the statement that a single ischemic preconditioning stimulus of 5 minutes global ischemia leads to the release of a sufficient number of humoral factors conferring strong cardioprotective effect in terms of infarct size reduction in the rat heart. Effluent collected after multiple IPC stimuli does not further enhance the infarct size reducing effect.
